# Gangrenous Grease, or Dermatitis Gangrenosa1Read before the Missouri Valley Veterinary Association, June, 1899.

**Published:** 1899-08

**Authors:** R. C. Moore

**Affiliations:** Kansas City, Mo.


### THE JOURNAL
OF
### COMPARATIVE MEDICINE AND VETERINARY ARCHIVES.

VOL. XX. *AUGUST, 1899.* No. 8.

### GANGRENOUS GREASE, OR DERMATITIS GANGRENOSA.[1]

BY R. C. MOORE, M.D.C.,
KANSAS CITY, MO.

So far as I have learned, Dollar's translation of *Moller's Surgery* contains the only mention of this serious and, at times, quite common malady. It is characterized by moist gangrene of the skin and adjacent tissues of the phalanges of solipeds, which produces extensive sloughing, and is supposed by some to be due to cold; but this idea is certainly incorrect. I have met with several cases, and not a single one in cold weather. On the contrary, it has developed during quite warm weather, and in at least one instance dry weather; so cold cannot be the sole cause. Infection through slight wounds is doubtless an important factor in the causation.

We might suspect obstruction to the circulation; but were that the cause the necrosis would be confined to the part that was robbed of nutrition, and the dead tissue would be separated from the living and no further invasion would occur; but in this affection new areas are rapidly invaded until the entire foot is destroyed or the animal dies from septic intoxication. It is evident that much remains to be learned of its etiology. It is sudden in its attack, often manifesting itself in the night, although its true character may remain obscure for one or two days.

The leg involved is swollen and extremely painful, and resembles an acute attack of scratches. In the latter the soreness and swelling subside with exercise, while in the former they do not, but are aggravated, and the debility of the patient is more marked.

[1] Read before the Missouri Valley Veterinary Association, June, 1899.

After a few hours a careful examination will reveal a moisture of the skin in the affected part, and by gentle pressure a red turbid serum can be squeezed out that has the characteristic odor of gangrene, the surface of the necrotic spots will be cold and clammy, while surrounding parts may have a supernormal temperature. The necrotic patches are usually small, but may involve larger areas. If located about the posterior part of the fetlock it may extend across the region as a crack, like scratches, or it may extend up and down on either side of the flexor tendon from the coronet to fetlock, or upward from the fetlock, involving the entire distal metacarpal region. The swelling may extend to the hock or knee, or even higher, the animal becomes restless, showing extreme pain. The temperature is elevated, the pulse accelerated, small and weak, and debility soon becomes well marked. In two or three days the necrotic patches are cast off as slimy masses. The disease may terminate here and the wound fill with granulations, and recovery by cicatrization be complete in two weeks; but this termination is the exception rather than the rule, for in most cases new cutaneous areas and as well the deeper structures are invaded, sloughing extends to bloodvessels, causing serious hemorrhages; tendons and ligaments are destroyed—even the capsular ligament, resulting in open-joint; or it may extend behind the lateral cartilage, resulting in cartilaginous quittor. It is not often that these conditions are found, excepting the destruction of the bloodvessels, as in most instances the system ere this absorbs a sufficient amount of poison to produce a general septic condition that rapidly leads to death, the difference in the termination probably being due to the ability of the system to resist the poison. When the patient has sufficient vitality to resist the generalization of the poison the destructive process extends to the deeper tissues of the affected limb, and the worst appearing sore that can well be imagined will be the result. The vessels, nerves, tendons, and ligaments having more power of resistance than the cellular tissue surrounding them, the latter is destroyed, leaving the former in position, so that they are often plainly visible for several inches; even the bone may become exposed to view. The coats of the veins being the thinnest, are usually the first to be destroyed after the cellular tissue, and serious, if not fatal, hemorrhage results.

The prognosis depends very largely on the extent and character of the individual case. It is claimed by some that certain atmospheric conditions favor gangrene, and during such season many wounds are troublesome, and at such times we would naturally expect gangrenous dermatitis to be more malignant. If the attack be mild and the invasion limited prognosis is favorable, but if it is extensive and rapidly extending to the deeper structures it is doubtful, and if ligaments, vessels and tendons are destroyed, or generalized septicæmia is established, the gravity of the case is greatly increased.

TREATMENT. As the infection may take place through a very small wound, we will not be likely to succeed by preventive measures. The disease being rapid in its progress, treatment in the advanced stages is doubtful, and if successful the time required to cicatrize the extensive wound is so long that the financial benefits to be derived by the owner from treatment is doubtful. Whatever therapeutic efforts are undertaken should be heroic and applied at the very earliest moment possible after the disease begins. An antiseptic course, locally as well as internally, is the one the conditions would most likely prompt us to have recourse to. I have frequently resorted to these without effect. I have gone further with germicides, even to a degree of potential cautery, and still with doubtful results.

The one agent which has been successful with me in this as well as other forms of gangrene is the actual cautery. I have treated these cases a number of times with the thermo-cautery when it seemed the entire foot was in an advanced stage of moist gangrene, even when sensation was so far destroyed that the animal would allow the hot iron to remain in the tissues for some time without the least resistance, and when I thought treatment was entirely useless, and twenty-four hours later would find the disease not only in check, but every trace of gangrene gone, leaving only the resulting sores, that required nothing more than the usual treatment for simple wounds.

Various kinds of firing-irons may be used. The thermo-cautery point that I have used is oval in shape, the largest diameter at the base being about one-half inch and tapering slightly to a blunt point. I prefer to use it at quite a high temperature, even a white heat, and to puncture into the deeper tissue in various directions, in all the invaded tissues, being careful not to wound the important vessels and joints.

Where the skin has sloughed the cautery point at nearly a white heat is applied to all the denuded surface with deep punctures around the edges. If the disease has originated in the sole from a wound, as may happen, and has extended up to the coronary band and adjacent tissues, I remove all the sole and frog that has been loosened by the disease, and if the sensitive sole is much diseased puncture it even to the solar surface of the os pedis. Twenty-four hours after firing, if it has been sufficient, the previously moist skin will be dry, and the underlying tissue, that has been soft and pulpy from infiltrated serum, will be reduced in size and be firm to the touch, with increased heat of the surface, and will be soon followed by a healthy discharge and granulation. If, at the expiration of the twenty-four hours, some of the parts are still discharging red turbid serum, are moist, cold, and clammy, more firing is indicated.

After the destructive process has been arrested, cleanliness, antiseptics, and astringents insure resolution. If the case be an aggravated one and debility is marked, stimulants are indicated, and the liberal use of hyposulphite of soda will assist in arresting the fermentation of the blood.

### *Discussion.*

Dr. Bennett: About how long was it after firing these cases till they recovered?

Dr. Moore: That will depend upon the amount of sloughing that has taken place. Usually twenty-four hours after firing.

Dr. Bennett: Do you think it is due to some specific cause?

Dr. Moore: I believe it is.

Dr. Bennett: You did not notice it in wet weather?

Dr. Moore: Yes.

Dr. Bennett: Was it more prevalent in dry weather?

Dr. Moore: I believe it is more prevalent in some stables than in others.

Dr. Bennett: Do you suppose that this is due to the unhealthful condition of the stable?

Dr. Moore: I do not know; I could not say that. The stables that I found it in were not particularly unhealthful.

Dr. Bennett: What was the condition of the animals you have noticed?

Dr. Moore: Some have been horses in good condition; some were hard-worked animals. I have met with these conditions ever since I have been in practice, but never understood it. Moller has given the first light I have been able to obtain on it. I do not believe it is confined entirely to heavy horses, yet I think we find more of it among them—probably there is more cause in that class of horses. I think the larger majority of the cases was in Clyde horses or in horses of a similar build. Referring to horses that have worked in coal-wagons, while I have seen one or two cases of that kind, yet I have seen one case in particular in an animal that had been in town only a short time in the spring of the year, and had not been worked more than a day or two. I do not believe that these conditions have very much or anything to do with it. I think it is infectious entirely.

